# Mathematical models for devising the optimal SARS-CoV-2 strategy for eradication in China, South Korea, and Italy

**DOI:** 10.1186/s12967-020-02513-7

**Published:** 2020-09-05

**Authors:** Shuo Jiang, Qiuyue Li, Chaoqun Li, Shanshan Liu, Xiaomeng He, Tao Wang, Hua Li, Christopher Corpe, Xiaoyan Zhang, Jianqing Xu, Jin Wang

**Affiliations:** 1 Shanghai Public Health Clinical Center, Fudan University, 2901 Caolang Road, Jinshan District, Shanghai, 201508 People’s Republic of China; 2 Wuhan Academy of Social Science, Wuhan, Hubei China; 3 State Key Laboratory for Oncogenes and Bio-ID Center, School of Biomedical Engineering, Shanghai Jiao Tong University, Shanghai, China; 4 King’s College London, Nutritional Science Department, 150 Stamford Street, Waterloo, SE19NH London, UK

**Keywords:** COVID-19, SARS-CoV-2, Mathematical models, Hospital isolation

## Abstract

**Background:**

Coronavirus disease 2019 (COVID-19), which is caused by severe acute respiratory syndrome coronavirus 2 (SARS-CoV-2), spreads rapidly and has attracted worldwide attention.

**Methods:**

To improve the forecast accuracy and investigate the spread of SARS-CoV-2, we constructed four mathematical models to numerically estimate the spread of SARS-CoV-2 and the efficacy of eradication strategies.

**Results:**

Using the Susceptible-Exposed-Infected-Removed (SEIR) model, and including measures such as city closures and extended leave policies implemented by the Chinese government that effectively reduced the *β* value, we estimated that the *β* value and basic transmission number, *R*_*0*_, of SARS-CoV-2 was 0.476/6.66 in Wuhan, 0.359/5.03 in Korea, and 0.400/5.60 in Italy. Considering medicine and vaccines, an advanced model demonstrated that the emergence of vaccines would greatly slow the spread of the virus. Our model predicted that 100,000 people would become infected assuming that the isolation rate *α* in Wuhan was 0.30. If quarantine measures were taken from March 10, 2020, and the quarantine rate of *α* was also 0.3, then the final number of infected people was predicted to be 11,426 in South Korea and 147,142 in Italy.

**Conclusions:**

Our mathematical models indicate that SARS-CoV-2 eradication depends on systematic planning, effective hospital isolation, and SARS-CoV-2 vaccination, and some measures including city closures and leave policies should be implemented to ensure SARS-CoV-2 eradication.

## Introduction

The outbreak of COVID-19 pneumonia in Wuhan, caused by the novel coronavirus SARS-CoV-2, has drawn tremendous attention around the world [1]. The ongoing COVID-19 outbreak resulted in more than 16,171,000 SARS-CoV-2 infections and over 647,350 deaths worldwide by July 25, 2020 [2]. SARS-CoV-2 has never been found in humans before and may not be as virulent as severe acute respiratory syndrome (SARS), but in humans, it is highly infectious.

Coronaviruses (CoVs) are pathogens that can infect the respiratory, gastrointestinal, hepatic and central nervous systems of humans, livestock, birds, bats, mice and other wild animals [3, 4]. Regarding the outbreaks of SARS in 2002 and Middle East respiratory syndrome (MERS) in 2012, the possibility of SARS-CoV and MERS-CoV transmission from animals to humans has been suggested [5, 6]. However, there have been no effective strategies, including therapeutics and vaccines, identified and the best approach to deal with severe CoV infections is to control the source of infection, using early diagnosis, isolation, and treatments and the timely dissemination of epidemic-related information to avoid unnecessary panic. Thus, surveillance and outbreak response management systems as a framework for COVID-19 outbreak modeling are urgently needed to control SARS-CoV-2 outbreaks worldwide.

Mathematical modeling plays an important role in understanding the complexities of infectious diseases and their containment [7] because it can rapidly meet the need for assessing the potential long-term impact of such diseases and offer strategies for the evaluation and prediction of the effect of possible interventions, even when available data are limited. Typical examples are the abundance of early models for HIV [8], pandemic influenza [9, 10], bovine spongiform encephalopathy (BSE) [11], and Creutzfeldt–Jakob disease (CJD) [12]. Since the outbreak of SARS, mathematical models have been published [13, 14], and policy makers have learned how models can help support their decision making. Based on the SARS-CoV-2 full genomic sequence data released on January 22, 2020 [15], that showed that SARS-CoV-2 has a more than 82% identical genome to those of SARS-CoV and bat SARS-like coronavirus (SL-CoV) [16], COVID-19 can now be tracked in the population, and rapid and accurate mathematical models can aid epidemiologic monitoring.

To improve forecast accuracy and investigate the spread and eradication pathways of SARS-CoV-2, a mathematical model needs to take into consideration patient improvements, personal protection strategies, regulation implementation, and other contributing factors. SIR (Susceptible-Infected-Removed) models the behavior of the early spread of infectious diseases and is generally used for measles, mumps, rubella and other infectious diseases [17]. Susceptible-Exposed-Infected-Removed (SEIR) model is similar to SIR, with the variables (S, I, and R) representing the number of people in each compartment at a particular time, but the incubation period has been added in SEIR so that it is more applicable to infectious diseases with a certain incubation period [18]. By considering the characteristics of SARS-CoV-2-its spread trends and local condition constraints-and economic optimization, we aimed to develop mathematical models that would provide the optimal COVID-19 eradication plan that was sensible and feasible.

## Methods

### Characteristics of SARS-CoV-2

To ensure the feasibility and usefulness of our approach and before constructing our models, the important attributes of SARS-CoV-2 were summarized. (1) Origin: according to the current etiological research, the natural host may be bats; the intermediate host is currently unknown. Most of the original patients were geographically linked to the Huanan Seafood Wholesale Market [19]. (2) Transmission: the route of SARS-CoV-2 infection is through direct, aerosol and contact transmission [20]. The CoV causing the current outbreak is different from the human coronaviruses previously identified, and the common ancestor of SARS/SARS-like coronaviruses is a virus similar to HKU9-1. The incubation period of SARS-CoV-2 is 1–14 days [4, 21–23].

### Assumptions of the model for SARS-CoV-2 analysis

Basic assumptions are that the outbreak began in Wuhan. After the outbreak, the city was closed on January 23, 2020, the remaining total population of Wuhan was 9,000,000, and we assumed it remained unchanged. The basic assumptions for South Korea and Italy are as follows: (1) The date that the first case occurred in China was Dec 08, 2019, and the date that the first case was diagnosed was Jan 11, 2020, with a difference of 34 days. We assumed that the actual occurrence of the first case in other countries was 30 days earlier than the first announced diagnosis. (2) In 2018, South Korea had a population of 51,640,000, and Italy had a population of 60,430,000.

### Four models for the numerical prediction of the spread of SARS-CoV-2

The datasets generated during and/or analyzed during the study of the model for SARS-CoV-2 are available on the following websites: (https://www.msn.com/en-gb/weather/other/coronavirus-outbreak-who-names; https://ncov.dxy.cn/ncovh5/view/pneumonia?from=timeline&isappinstalled=0; and http://www.zq-ai.com/#/fe/xgfybigdata). All information on the basic considerations and assumptions of the SIR and SEIR models are included in the “Methods” section and Additional file 1 provide more information about our models. All related parameters are shown in Table 1. For the SIR model, the sensitivity of the *β* parameter (the probability of transfer from the susceptible state to the resistant state) was estimated. There was *N* ≈ *S* in the early stage of the epidemic, so: .

$$ \frac{dI}{dt} = \beta \frac{IS}{N} - \gamma I \approx \left( {\beta - \gamma } \right)I $$

**Table 1:** Parameters of our mathematical models

Parameters	Symbol	Description
Susceptible group	*S*	People who have no immunity against the disease. They are very likely to be infected by coming in direct contact with infected people
Exposed group	*E*	People who have been infected but have not displayed any explicit symptoms. They do not transmit the virus to susceptible people
Infected group	*I*	People in the infected group show explicit symptoms of SARS-CoV-2, and they can transmit the virus to susceptible people
Removed group	*R*	The removed group includes people who have died of the disease or who have survived the disease. People who have survived the disease will obtain complete immunity against it
Number	*N*	The total population
Infection rate	*β*	The probability that a susceptible person will become ill after coming into contact with an infected person
Outflow rate	*σ*	The outflow rate of the incubation group to the infected group
Period	*γ*	The days from infected to removed
Isolation rate	*α*	The rate of people moving from the infected group to the hospital isolated group
Outflow rate	*ω*	The outflow rate of the infected group to the hospital isolated group
Vaccination rate	*θ*	The percentage of susceptible people who receive a vaccination each day
Number	*H*	The number of people in the hospital isolation group
Number	*I*_*E*_	The number of people in the early infection group
Number	*I*_*L*_	The number of people in the advanced infection group
Period	*γ*_*E*_	Period when patients in the early infected group display explicit symptoms of COVID-19 and can transmit the virus to susceptible people
Period	*γ*_*L*_	Period when patients in the advanced infected group display explicit symptoms of COVID-19 and can transmit the virus to susceptible people
Number	*M*	The number of people in the immunity group

Hence: .

$$ I\left( t \right) = e^{{\left( {\beta - \gamma } \right)t}} $$

For the SEIR model, we divided the total population into four groups, including the Susceptible (*S*), Incubation (*E*), Infected (*I*) and Removed (*R*) groups, and modeled SARS-CoV-2 infection as related to the number of instances of contact between susceptible and infected people multiplied by the infection rate *β*. We can express the rate of change of the susceptible group as:

$$ \frac{dS}{dt} = - \beta {\kern 1pt} {\kern 1pt} S{\kern 1pt} {\kern 1pt} \frac{I}{N} $$

Note that *σ* represents the outflow rate of the incubation group to the infected group. The rate of change of the incubation group can be expressed as:

$$ \frac{dE}{dt} = \beta {\kern 1pt} {\kern 1pt} S{\kern 1pt} {\kern 1pt} \frac{I}{N} - \sigma E $$

As *γ* represents the outflow rate of the infected group to the removed group, we obtain the following equation:

$$ \frac{dE}{dt} = \beta {\kern 1pt} {\kern 1pt} S{\kern 1pt} {\kern 1pt} \frac{I}{N} - \sigma E $$.

We can obtain the rate of change of the removed group. Its inflow is from the infected group at the rate of *γ,* and it has no outflow since it is the end of the system. Hence, we obtain the following equation:

$$ \frac{dR}{dt} = \gamma I. $$

The whole process set out above can be displayed in the flow chart below.
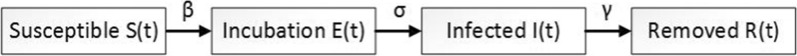


Furthermore, we made some modifications to the basic model by including hospital isolations. The outflow rate of the infected group will change since part of this group will be moved to the hospital isolation group at the rate of *α,* and the other part will move to the removed group at the rate of *γ*. Hence, we have:

$$ \frac{dI}{dt} = \sigma E - \gamma (1 - \alpha )I - \alpha I $$.

For the new hospital isolation group, its inflow is from the infected group at the rate of *α*, and its outflow is to the removed group at the rate of *ω*. We can obtain the rate of change of this group:

$$ \frac{dH}{dt} = \alpha I - \omega H $$

Similarly, we made some modifications to the rate of change of the removed group:

$$ \frac{dR}{dt} = \gamma (1 - \alpha )I + \omega H $$

We next define *α* as the isolation rate, which is the rate of people moving from the infected group to the hospital isolated group, and *ω* as the outflow rate of the infected group to the hospital isolated group. Let *H* be the number of people in this group. The advanced model introduced above can be displayed in the flow chart below.
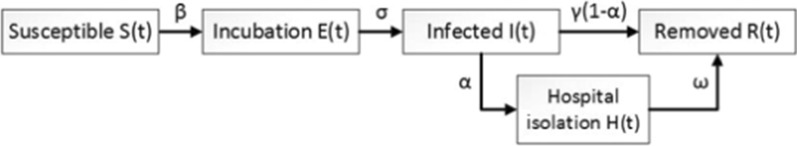


Finally, we incorporated effective medications and vaccines into this advanced model. We define *M* as the number of people in the immunity group, *I*_*E*_ as the number of people in the early infection group, and *I*_*L*_ as the number of people in the advanced infection group. We present the system of differential equations for our advanced model considering medication and vaccination as follows:

$$ \begin{aligned} \frac{dS}{dt} = - \beta S\frac{{I_{E} + I_{L} }}{N} - \theta S \hfill \\ \frac{dE}{dt} = \beta S\frac{{I_{E} + I_{L} }}{N} - \sigma E \hfill \\ \frac{{dI_{E} }}{dt} = \sigma E - \gamma_{E} (1 - \alpha )I_{E} - \alpha I_{E} \hfill \\ \frac{{dI_{L} }}{dt} = \gamma_{E} (1 - \alpha )I_{E} - \gamma_{L} I_{L} \hfill \\ \end{aligned} $$.

$$ \begin{aligned} \frac{dH}{dt} = \alpha I_{E} - \omega H \hfill \\ \frac{dM}{dt} = \theta S + \omega H \hfill \\ \frac{dR}{dt} = \gamma_{L} I_{L} \hfill \\ \end{aligned} $$

The above model can be displayed in the flow chart shown below.
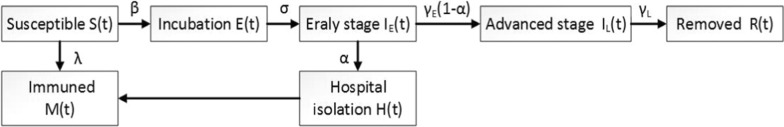


## Results

### Disease review of COVID-19

**Fig. 1:**
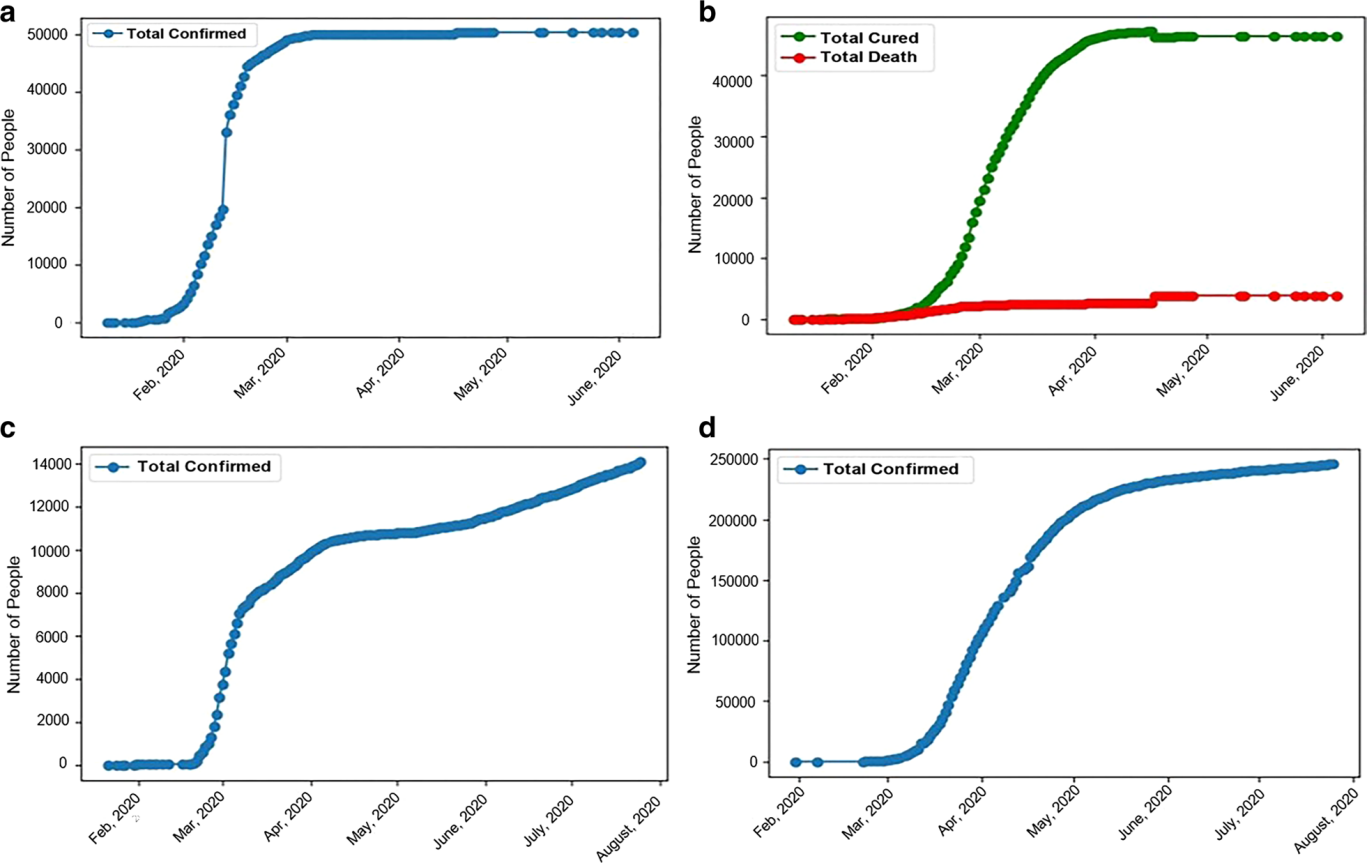
Review of COVID-19 in Wuhan, Republic of Korea, and Italy. **a** The number of confirmed cases in Wuhan; **b** the number of cumulative cured patients and deaths in Wuhan; and the number of confirmed cases in South Korea (**c**) and Italy as of July 25, 2020 (**d**)

The first confirmed case of COVID-19 was on Dec 8, 2019, and the Chinese government began to continuously report cases on Jan 11, 2020. After the early outbreak of SARS-CoV-2, as of March 4, 2020, 80,424 cases had been confirmed in China, including 2,984 deaths [24]; outside China, 10,566 cases were reported in 72 countries, and a large percentage of those cases occurred in South Korea and Italy (2). Figure 1 shows the detailed epidemic trend until July 25, 2020, and includes the number of confirmed cases in Wuhan (Fig. 1a); Fig. 1b shows the number of cumulative treated and recovered patients and the number of deaths in Wuhan. Detailed epidemic trends that include the number of confirmed cases in South Korea (Fig. 1c) and Italy (Fig. 1d) are also shown. The severity of the situation means an accurate mathematical model needs to be established to predict epidemic trends in order to take positive and effective countermeasures. The actions these affected countries take today will be the difference between a handful and a larger cluster of cases.

### Effect of the SARS-CoV-2 infection rate on eradicating SARS-CoV-2

**Fig. 2:**
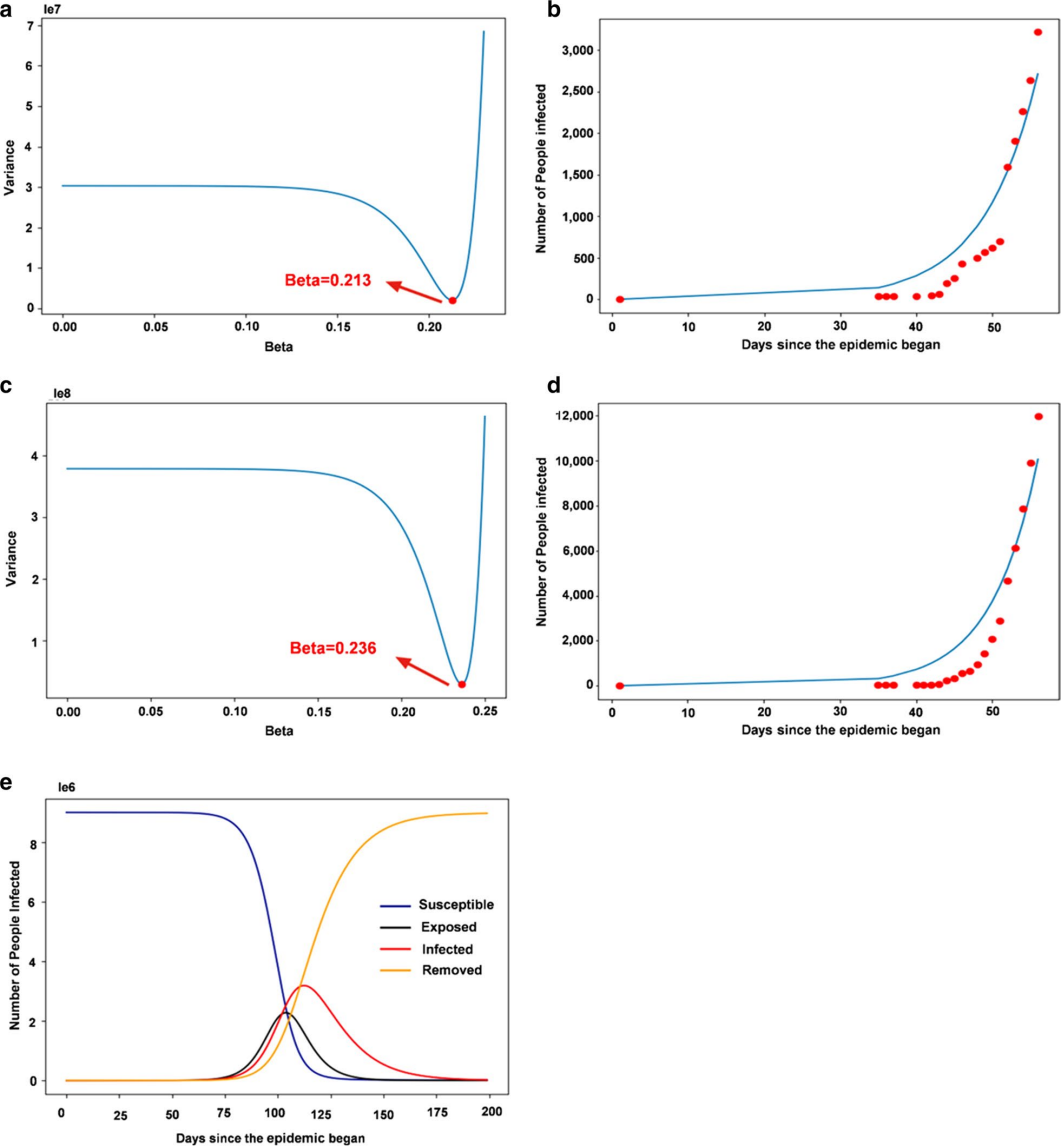
The SIR and SEIR models analyze the basic epidemic data from Wuhan: **a, b** optimistic estimation and **c, d** pessimistic estimation. **a** The *β* value in the optimistic estimation. **b** The infection cases in the optimistic estimation are reasonable. **c** The *β* value in the pessimistic estimation. **d** The fitting of infection cases in the pessimistic estimation. **e** Trends in each group of SEIR models for Wuhan

Under the optimistic estimation, the number of cases reported in only Wuhan is counted as the confirmed cases recorded in Wuhan. In the modeling, according to the pessimistic estimate, it is assumed that the infection cases found in other areas are all from Wuhan. By iterating on different *β* values, the *β* value with the minimum variance is selected as the fitted *β* value:

$$ \mathop {\hbox{min} }\limits_{\beta } \sum\limits_{i = 1}^{n} {(f_{i} - d_{i} )^{2} } $$.

where $f_{i}$ denotes the data *i* on the fitted curve, $d_{i}$ denotes the real data and *n* denotes the data size. It can be concluded that the *β* value in the optimistic case is 0.213 (Fig. 2a). Therefore, the fitting of infection cases under optimistic conditions is shown in Fig. 2b. The pessimistic estimation indicates that the *β* value in the pessimistic case is 0.236 (Fig. 2c). Therefore, the fitting of infection cases under pessimistic conditions is shown in Fig. 2d. It can be concluded that the value of the resistance parameter (the probability of transfer from one susceptible individual to another) is between 0.213 and 0.236. We can also estimate the basic reproduction number (*R*_*0*_) of the novel coronavirus as:

$$ R_{0} = \frac{\beta }{\gamma } = \frac{0.213 \sim 0.236}{1/14} = 2.98\sim 3.30 $$.

The *R*_*0*_ of infections is the average number of people infected with an infectious disease that can spread to other people without intervention and without immunity. The larger the *R*_*0*_ number is, the more difficult it is to control the epidemic. In this model, we estimated that the basic transmission *R*_*0*_ of SARS-CoV-2 was between approximately 2.98 and 3.30.

### Effect of SARS-CoV-2 government control measures on eradicating SARS-CoV-2

**(**1) In Wuhan: As of Feb 12, 2020 (day 67), a total of 19,558 cases were confirmed in Wuhan. That is, I (67) = 19,558 can be solved as *β* = 0.476. In the SEIR model, the *R*_*0*_ of the novel coronavirus is:

$$ R_{0} = \frac{\beta }{\gamma } = \frac{0.476}{1/14} = 6.66 $$.

**Fig. 3:**
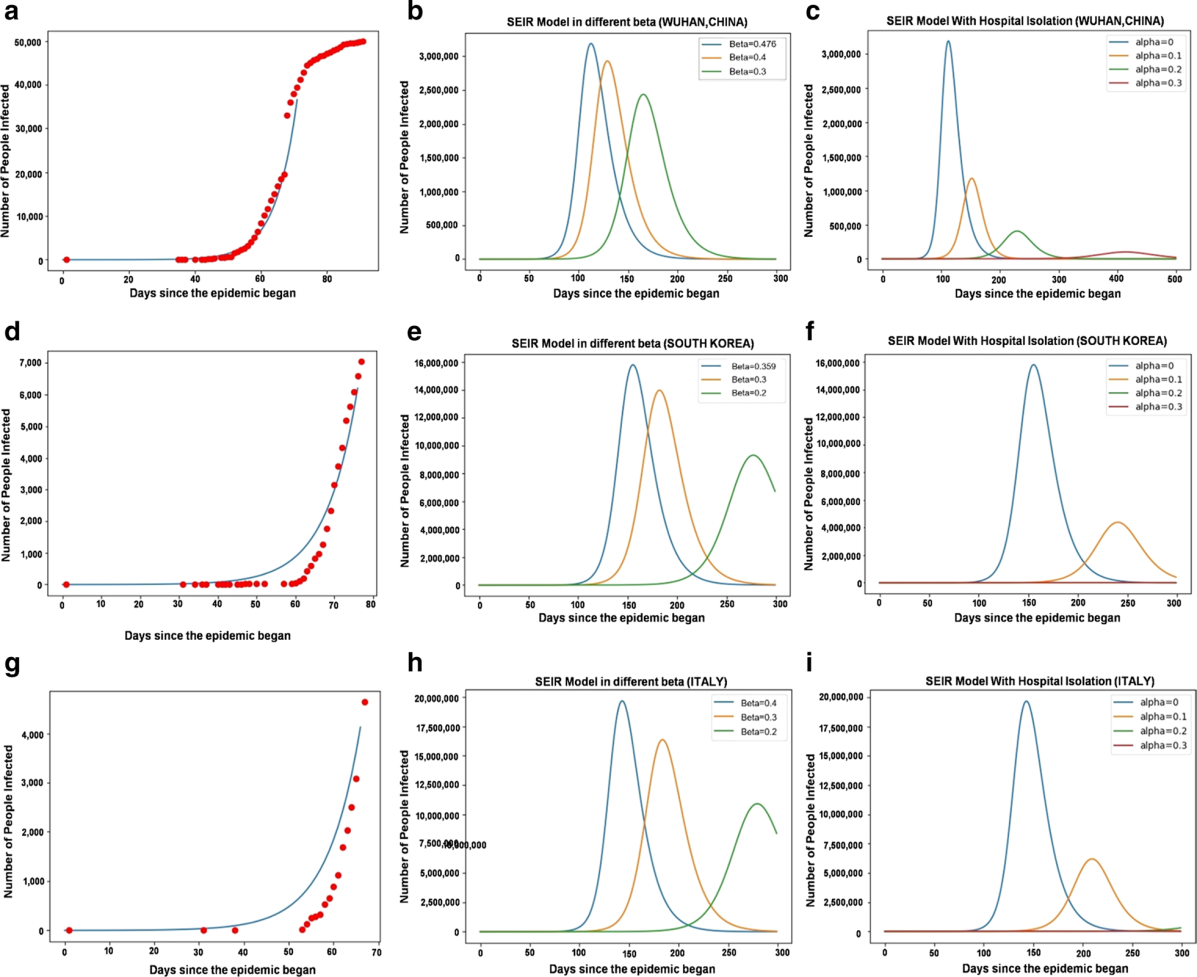
The SIR and SEIR models were used to analyze the epidemic situation in Wuhan. **a** The fitted curve (blue) for Wuhan, where red points represent the actual number of cases; **b** SEIR model analysis for Wuhan; and **c** Hospital isolation included in the model analysis for Wuhan. Hospital isolation model with different isolation ratios showing that the peak of the outbreak occurred on day 450. **d** The fitted curve (blue) for South Korea; red points represent the actual number of cases. **e** SEIR model analysis for South Korea. **f** Hospital isolation model with different isolation ratios included in the model analysis for South Korea. The peak of the outbreak occurred on day 450. **g** The fitted curve (blue) for Italy; red points represent the actual number of cases. **h** SEIR model analysis for Italy. **i** Hospital isolation model with different isolation ratios included in the model analysis for Italy, and the peak of the outbreak occurred on day 405

It can be found that the disease outbreak starts to intensify around the 80th day (late Feb) and reaches its peak around the 120th day (mid-April). The changing trend of each group is shown in Fig. 2e. According to the actual number of cases in Wuhan, the epidemic curve was fitted (Fig. 3a), and *β* values were evaluated. For government control of the outbreak, under the SEIR model, we simulated that *β* was 0.476 (Fig. 3b). According to the above *β* value, we estimated that approximately 3,200,000 people will be infected in the whole city of Wuhan. When *β* decreases to 0.4, the peak infection rate drops to 3,000,000, and when *β* further decreases to 0.3, the peak infection rate drops to 2,500,000 (Fig. 3b).

(2) In South Korea and Italy: In the SEIR model, the basic reproduction number of the novel coronavirus in South Korea and Italy is:

$$ R_{0} \left( {South Korea} \right) = \frac{\beta }{\gamma } = \frac{0.359}{1/14} = 5.03 $$;

$$ R_{0} \left( {Italy} \right) = \frac{\beta }{\gamma } = \frac{0.400}{1/14} = 5.60 $$.

According to the actual number of cases in Korea and Italy, the epidemic curve was fitted (Fig. 3d, g), and *β* values were evaluated. For government control of the outbreak, under the SEIR model, we simulated that *β* was 0.359 for South Korea (Fig. 3e) and 0.400 for Italy (Fig. 3h). According to the above *β* values, we estimated that approximately 51,640,000 and 60,430,000 people would be infected in South Korea and Italy, respectively. If the government implements some measures, such as city closure policies and extended leave policies, it can effectively reduce the *β* value. In South Korea, when *β* drops to 0.30 or 0.20, the peak infection rate drops to 13,974,030 or 9,313,317 (Fig. 3e). In Italy, when *β* decreases to 0.30 or 0.20, the peak infection rate drops to 16,358,690 or 10,897,254 (Fig. 3h).

### Effect of hospital isolation on eradicating SARS-CoV-2

Applying different *α* ratios, we can see that with the increase in the ratio of hospital isolation, the number of infections at the highest point decreased, and the time of peak occurrence was later. When not isolated, approximately 3,200,000 people eventually become infected in Wuhan. When only 10% of the population is quarantined, only 1,200,000 become infected in Wuhan. When the quarantine rate is raised further, to 20% or 30%, 400,000 or only 100,000 people, respectively, would become infected (Fig. 3c), which is consistent with the current number of infected people in the report, verifying that 30% of the population has been quarantined in Wuhan according to this modeling analysis. Additionally, if quarantine measures were implemented from March 10, 2020, and the quarantine rate of *α* was 0.3, the final number of infected people would be 11,426 in South Korea with a peak of 450 days (Fig. 3f) and 147,142 in Italy with a peak of 405 days (Fig. 3i).

### Effect of the vaccination rate and time on eradicating SARS-CoV-2 in Wuhan

**Fig. 4:**
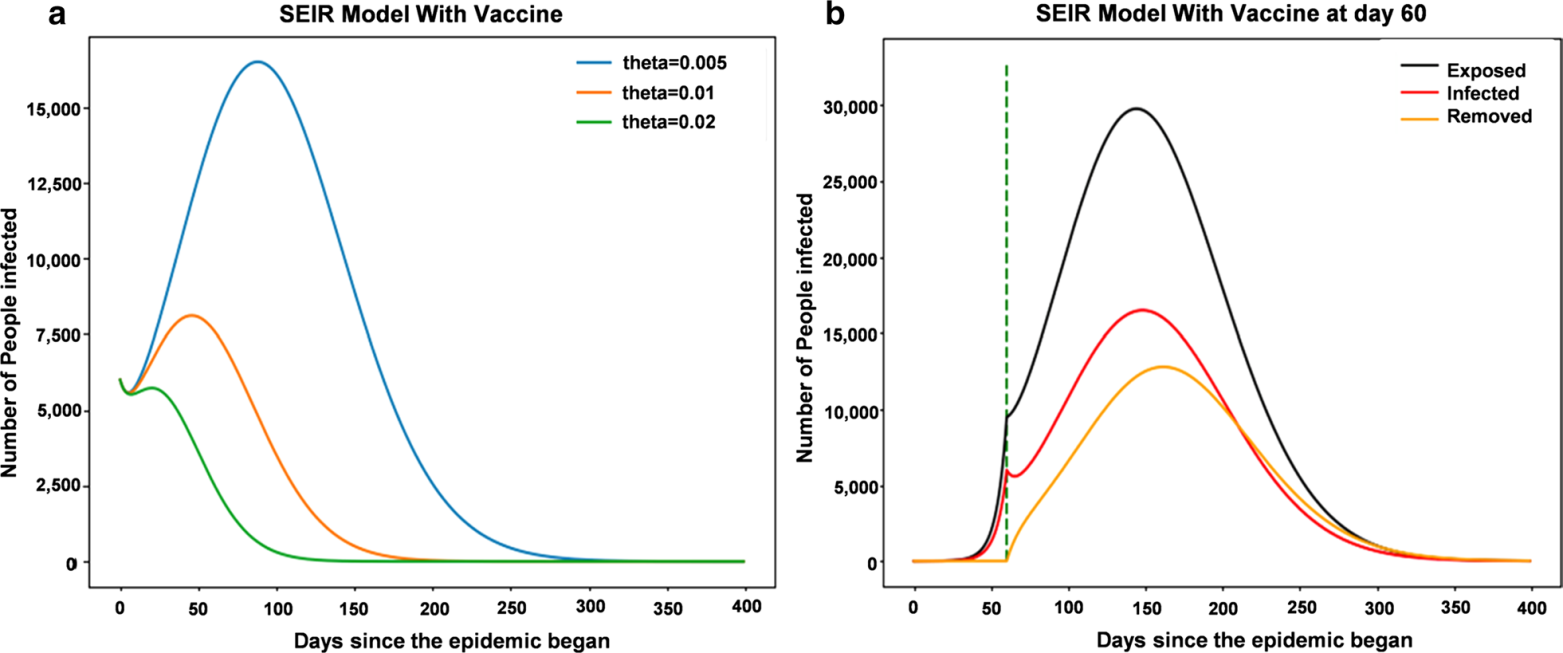
Vaccination rates were included in the model analysis for Wuhan. **a** SEIR model with different vaccination rates. **b** The changes of the SEIR model with vaccination starting on day 60

Vaccination rate: Assuming the isolation rate alpha is 0.2, the changes in the number of infected patients under different vaccination rates (theta) are shown in Fig. 4a. Without a vaccine, at a 20% isolation rate, 400,000 people could be infected. If the vaccination rate is 0.005, fewer than 20,000 people will become infected. Therefore, the emergence of vaccines can greatly alleviate the spread of a virus. If a vaccine is developed within 2 months of the outbreak (day 60) with a vaccination rate of 0.005, the changes after that are shown in Fig. 4b; this implies that the SARS-CoV-2 will peak at day 150 and only 15,000 people in Wuhan will be infected. However, it will take a few months to develop a vaccine for SARS-CoV-2.

## Discussion

To date, the COVID-19 epidemic is still in a phase of rapid dispersion worldwide, and this epidemic represents a clear and ongoing global health threat. It is currently uncertain whether it is possible to contain the continuing epidemic within China [25]. In the early stage of a SARS-CoV-2 outbreak, the medical conditions are not ideal, and no effective measures have been taken. The number of infected people increases exponentially, so it is particularly critical to control the value of *β*. In our model, the *β* of South Korea is 0.359, which is the lowest among the three countries, followed by Italy, whose *β* is 0.4. The *β* of both South Korea and Italy is lower than that of China. In these three countries, the trend of *R*_*0*_ was consistent with *β*. Thus, reducing the exposure rate of *β* could significantly reduce the reproduction number. Next, we estimated an *R*_*0*_ of 6.66 in Wuhan, which represents a relatively higher value than those computed so far (as of Mar 01, 2020). In several other mathematical models that have been devised and released to date, *R*_*0*_ varies from 1.30 to 6.47 (Additional files 1, 2: Table S1) [26–30]. By comparing the methodologies of the various investigations, these different reproduction numbers reflect the dynamics of transmission, and the cases of COVID-19 fluctuate and vary over time. In our models, we used these data (Jan 15, 2020–Mar 01, 2020) to estimate a relatively accurate *R*_*0*_, which was relatively reliable in our models.

We further took hospital isolation and SARS-CoV-2 drugs and vaccines into account in our models. With other parameters unchanged, the greater the *α* for the isolation rate in the hospital, the later the peak is reached, the smaller the peak size, and the more effective it is to prevent the spread of the disease. The hospital pathway should sufficiently isolate SARS-CoV-2-infected patients from other patients to decrease infection, and more concern should be given about protecting doctors and nurses [31]. For the penetration of vaccines and drugs, the greater the parameter theta, the earlier the peak would appear, the smaller the peak would be, and the lower the final total number of infections would be. However, scientists have still not found effective medications and vaccines for SARS-CoV-2 [32]. Finally, our simulations have shown that the containment outcome depends highly on the effectiveness of the intense control effort now underway in China. Reducing the exposure rate of *β* and increasing the isolation rate of *α* can significantly reduce the number of infected people. The government should continue to tightly monitor the epidemic situation and must take immediate measures against it, and this includes immediate isolation of newly infected people and closures of cities with severe outbreaks, in case there are unexpected outbreaks in the eradication process. Regional transmission is the root of the spread of COVID-19. Local governments must have the responsibility to set a deadline for the final eradication, and the SARS-CoV-2 epidemic in the world revealed that all the countries still need to strengthen the establishment of a rapid outbreak response strategy and health policies. Finally, there were some limitations in our SIR or SEIR models of SARS-CoV-2, such as we only used the average latency value during the latent period. We recognized that some patients with COVID-19 have a longer latent period, while others have a shorter latent period of only 4 days, which affects the basic reproduction number (*R*_*0*_) [33]. In addition, we need to consider “superspreading events and superspreaders”, which tend to occur at large gatherings with close contact [34], transmit infection to a larger number of individuals than is typical by one individual [35], and affect *R*_*0*_. For example, a man who later tested positive for COVID-19 visited several clubs in Seoul and infected 170 new individuals after South Korea relaxed social distancing rules in May. Moreover, the differences in case definitions and reporting measures, city closures and leave policies, and viral testing would likely affect the basic reproduction number and the secondary attack rate (SAR) [34, 36]. It is important that the Italian government implemented extraordinary measures to limit viral transmission in March 2020 and minimized the likelihood that people were infected [37]. Therefore, all those countries that had beaten back the virus to low levels need to be especially vigilant for superspreaders and superspreading events.

## Conclusions

In summary, our mathematical model of SARS-CoV-2 infection can accurately predict the incidence and number of cases as well as the peak and end times of the epidemic and provide feasible solutions for future epidemic prevention and control, including predicting future epidemic trends and providing a reference for effective control options. Our results emphasized that effective SARS-CoV-2 eradication must involve active cooperation between the government, pharmaceutical companies and hospital organizations.

## Supplementary information

**Additional file 1:** Additional methods of the basic considerations and assumptions.

**Additional file 2: Table S1.** Reports in the different mathematical model published in COVID-19.

## Data Availability

The datasets generated during and/or analyzed during the current study are available on the following websites: (https://www.msn.com/en-gb/weather/other/coronavirus-outbreak-who-names;https://ncov.dxy.cn/ncovh5/view/pneumonia?from=timeline&isappinstalled=0; and http://www.zq-ai.com/#/fe/xgfybigdata).
