# Supplementary material for: Mathematical models for devising the optimal SARS-CoV-2 strategy for eradication in China, South Korea, and Italy
Source: J Transl Med. 2020 Sep 5;18:345. doi: 10.1186/s12967-020-02513-7 (PMC7474336; doi:10.1186/s12967-020-02513-7)
Supplement: Supplementary file 1 — Additional file 1: Additional methods of the basic considerations and assumptions. [file 12967_2020_2513_MOESM1_ESM.docx]

**Additional methods of the basic considerations and assumptions.**

**1.1. Model 1: *SIR* model.**

**Basic Considerations.** Parameters obtained from market performance: (1) it takes an average of 7 days from Susceptible to Infected, so$\sigma$=1/7; (2) it takes an average of 14 days from Infected to Removed, so $\gamma$=1/14. Estimated of the β parameter sensitivity (the probability of transfer from the cultivated state to the resistant state): At the beginning, the number of infected persons was 1. We used the early stage of the pneumonia outbreak data to estimate the parameters.

**1.2. Model 2: *SEIR* model.**

**Basic Considerations.** In this study, we divided the total population into four groups (1) Susceptible group (*S*): People who have no immunity against the disease. They are very likely to be infected by coming in direct contact with infected people (see below). Let *S* denote the number of people in the susceptible group. (2) Incubation group (*E*): People who have been infected but have not displayed any explicit symptoms. They do not transmit the virus to susceptible people. Let *E* denote the number of people in the incubation group. (3) Infected group (*I*): People in the infected group show explicit symptoms of SARS-CoV-2, and they can transmit the virus to susceptible people. Let *I* denote the number of people in the infected group. (4) Removed group (*R*): The removed group includes people who have died of the disease or who have survived the disease. People who have survived the disease will obtain complete immunity against it. Let *R* denote the number of people in the removed group.

**Assumptions:** (1) There is currently no vaccine, so no one is naturally resistant to the virus; (2) the virus does not mutate; once a person is cured by his or her own immune system, the person will not get re-infected.

**1.3. Model 3:** **Advanced Model 1: Considering hospital isolation.**

**Basic Considerations.** We incorporated the hospital isolation factor in this model to fit the actual situation. The function of hospital isolation is to decrease the chances of contact between susceptible and infected people; therefore, the spread of the virus can be controlled down to a lower level. We needed to add one more group on the basis of the basic model and define some parameters for the new group. Hospital isolation group: People in this group are isolated from susceptible people. We define *𝛼* as the isolation rate, which is the rate of people moving from the infected group to the hospital isolated group. The larger *α* is, the higher the medical condition and the spread of SARS-CoV-2 can be expected to be at a lower level. We also define *ω* as the outflow rate of this group; thus, *1/ω* denotes the period that one person stays in this group. Let *H* be the number of people in this group.

**Assumptions:** (1) People in the incubation group are treated as susceptible people and will not be moved into hospital isolation; (2) People in the hospital isolation group have absolutely no chance to infect susceptible people; (3) People in the hospital isolation group will also die after a *1/ω* period of time since there is no medication in this model. We assume that *1/ω=1/γ,* that is, isolated people, will die with the same rate of infected people.

**1.4. Model 4: Advanced Model 2: Considering medicine and vaccine.**

**Basic Considerations.** We further incorporated drugs and vaccines into this advanced model. We first made some changes about the grouping of people based on advanced model one. **Immunity group:** People in this group gain complete immunity against SARS-CoV-2 virus. They can either obtain immunity through a vaccination or recover from the disease. Let *M* denote the number of people in this group, and *θ* denote the vaccination rate; that is, the percentage of susceptible people who receive a vaccination each day. **Infected group:** These people should be further divided into two sub-groups in this model. **Early infected group:** People who have displayed explicit symptoms of COVID-19 and can transmit the virus to susceptible people. In addition, they can be cured with COVID-19 drugs. We assume this phase lasts 3 days. Let *I_E_* denote the number of people in this group and *1/𝛾_E_* denote the period of this phase. **Advanced infected group:** People who also have displayed explicit symptoms of COVID-19 and can transmit the virus to susceptible people. However, they cannot be cured by the COVID-19 drugs. We assume this phase lasts 2.6 days. Let *I_L_* denote the number of people in this group and *1/𝛾_L_* denote the period of this phase.

**Assumptions:** (1) Medications, including vaccines and drugs, have been used since February 1, 2020. In addition, we set this day as t = 0; (2) Vaccines have no effect on people in the incubation group, i.e., vaccines can either provide immunity or cure the disease for them; (3) Infected people are only moved to hospital isolation during the early infected phase. Advanced infected people will not enjoy the same treatment; (4) Drugs are only for people in hospital isolation; (5) Each person in the hospital isolation group will be provided with enough medications so that they will all recover from the disease. The drug production quantity is sufficient.
