# Supplementary material for: Mathematical models for devising the optimal SARS-CoV-2 strategy for eradication in China, South Korea, and Italy
Source: J Transl Med. 2020 Sep 5;18:345. doi: 10.1186/s12967-020-02513-7 (PMC7474336; doi:10.1186/s12967-020-02513-7)
Supplement: Supplementary file 2 — Additional file 2: Table S1. Reports in the different mathematical model published in COVID-19. [file 12967_2020_2513_MOESM2_ESM.docx]

**Table S1. Reports in the different mathematical model published in COVID-19.**

| **Models** | **Methods** | **Parameters and results** | **References** |
| --- | --- | --- | --- |
| GLEAM | BCA | *T_g_*: 7.5 days; *R_0_*: 2.57 (90% CI 2.37 - 2.78); *T_d_*: 4.2 days (90% CI 3.8 - 4.7) | (1) |
| SEIR model | MCMC method | *R_0_*: 2.68 (95% CrI 2.47-2.86); The epidemic doubling time: 6.4 days (95% CrI 5.8-7.1) | (2) |
| A branching process model | A negative binomial distribution | *R_0_*: 1.5, 2.5, 3.5 | (3) |
| Stochastic simulations model | A negative-binomial offspring distribution | *R*_0_ was 2.2 (90% high density interval: 1.4 - 3.8); *k*: median: 0.54, 90% high density interval: 0.014-6.95 | (4) |
| SEIR model | --- | *R*_0_ was 3.11 (95% CI, 2.39 - 4.13) | (5) |
| GAM | The lagged probability distribution | *R_0_* of nationwide and Wuhan: 4.5, 4.4; The doubling time: 2.4 days | (6) |
| Simple non-linear growth models | The NLS framework | *R*_0_: 2.24 (95% CI: 1.96 - 2.55) to 3.58 (95% CI: 2.89 - 4.39); g was 8- and 2-fold | (7) |
| A dynamic compartmental model (Eq. (1)-(2)) | NLS method; MCMC methods;  M-H algorithm | Basic *R_0_*: 4.71 (4.50 - 4.92); Effective *R_0_*: 2.08 (1.99 - 2.18); The epidemic peak time: peak in early March 2020 (80 days since initiation) | (8) |
| IDEA model | --- | *R_0_* varied from 2.0 to 3.1 | (9) |
| BDSS model | The Bayesian MCMC algorithm | The median estimate of *Re* shifted from 1.6 to 1.1 on around January 1, 2020. | (10) |
| SEIR and SEIHR models | The least square method; MCMC; Likelihood function method | *R_0_* was 6.47 (95% *CI*：5.71 - 7.23) | (11) |
| Modified SIR model | --- | The actual number of infected cases: 88,075 cases (Jan 31, 2020); Isolation wards and ICU were 34,786 and 9,346, respectively | (12) |

**Notes:** GLEAM: The Global Epidemic and Mobility Model; Bayesian Computation approach: BCA; *T_g_*: A generation time; *R_0_*: reproductive number; *T_d_*: a doubling time; MCMC: Markov Chain Monte Carlo; *k*: Dispersion parameter; GAM: generalized additive model; NLS: nonlinear least square; g: Intrinsic growth rate; MH: Metropolis-Hastings; IDEA: Incidence Decay and Exponential Adjustment; EG: Exponential Growth; ML: maximum likelihood; BDSS: birth-death skyline serial; *Re*: effective reproductive number

**References**

1. Chinazzi M, Davis JT, Ajelli M, et al. The effect of travel restrictions on the spread of the 2019 novel coronavirus (COVID-19) outbreak. *Science*. 2020;368(6489):395-400.

2. Wu JT, Leung K, Leung GM. Nowcasting and forecasting the potential domestic and international spread of the 2019-nCoV outbreak originating in Wuhan, China: a modelling study. Lancet. 2020;395(10225):689-97.

3. Hellewell J, Abbott S, Gimma A, et al. Feasibility of controlling COVID-19 outbreaks by isolation of cases and contacts. *Lancet Glob Health*. 2020;8(4):e488-e496.

4. Riou J, Althaus CL. Pattern of early human-to-human transmission of Wuhan 2019 novel coronavirus (2019-nCoV), December 2019 to January 2020. *Euro Surveill*. 2020;25(4):2000058.

5. Wan K, Chen J, Lu C, Dong L, Wu Z, Zhang L. When will the battle against novel coronavirus end in Wuhan: A SEIR modeling analysis. *J Glob Health*. 2020;10(1):011002.

6. Liu T, Hu J, Xiao J, He G, Kang M, Rong Z, et al. Time-varying transmission dynamics of Novel Coronavirus Pneumonia in China. bioRxiv. 2020:2020.01.25.919787.

7. Zhao S, Lin Q, Ran J, Musa SS, Yang G, Wang W, et al. Preliminary estimation of the basic reproduction number of novel coronavirus (2019-nCoV) in China, from 2019 to 2020: A data-driven analysis in the early phase of the outbreak. Int J Infect Dis. 2020;92:214-7.

8. Shen M, Peng Z, Xiao Y, Zhang L. Modelling the epidemic trend of the 2019 novel coronavirus outbreak in China. bioRxiv. 2020:2020.01.23.916726.

9. Majumder MS, Mandl KD. Early Transmissibility Assessment of a Novel Coronavirus in Wuhan, China. Preprint. *SSRN*. 2020;3524675.

10. Zhang C, Wang M. MRCA time and epidemic dynamics of the 2019 novel coronavirus. 2020.

11. Tang SY, Xiao YN, Peng ZH, Shen HB. *Zhonghua Liu Xing Bing Xue Za Zhi*. 2020;41(4):480-484.

12. Ming W-K, Huang J, Zhang CJP. Breaking down of healthcare system: Mathematical modelling for controlling the novel coronavirus (2019-nCoV) outbreak in Wuhan, China. bioRxiv. 2020:2020.01.27.922443.
